# The prescribing of opioids for chronic non-cancer pain in the menopausal and postmenopausal population: a drug utilisation study in the UK

**DOI:** 10.3399/BJGPO.2024.0169

**Published:** 2025-04-09

**Authors:** Emma Tillyer, Yogini Jani, Li Wei, Ruth Brauer

**Affiliations:** 1 Research Department of Practice and Policy, School of Pharmacy, University College London, London, UK; 2 Centre for Medicines Optimisation Research and Education, University College London Hospitals NHS Foundation Trust, London, UK

**Keywords:** women’s health, prescribing, pain, menopause

## Abstract

**Background:**

Opioid use for chronic non-cancer pain (CNCP) is consistently higher in menopausal and postmenopausal women than in younger women or men, elevating their risk of opioid-related adverse health outcomes. Since pain severity increases with hormonal changes accompanying menopause, these women should be a focus of opioid stewardship efforts.

**Aim:**

To examine opioid prescribing trends for CNCP in menopausal and postmenopausal women diagnosed with a musculoskeletal condition.

**Design & setting:**

Population-based drug utilisation study, which was undertaken with data from IQVIA Medical Research Data UK.

**Method:**

Annual opioid prescribing incidence, prevalence, and average duration of use were calculated for a cohort of women aged 50–79 years with musculoskeletal conditions newly diagnosed between 2010 and 2021. Specific results were stratified by age, pain indication, and Townsend score.

**Results:**

From 2010–2021, incident prescribing rates of opioids increased in women aged 50–54 years (161.4 [95% confidence interval {CI} = 149.7 to 174.0] per 1000 person–years at risk [PYAR] in 2010 to 239.6 [95% CI = 211.7 to 271.2] per 1000 PYAR in 2021); these women discontinued opioid use faster (<1 year) than older age groups (~2 years). Overall, opioid prescribing prevalence decreased from 23% in 2010 to 14% in 2021, and average opioid use duration decreased from 3 years to 1 year (2010–post-2017) in women aged 50–79 years.

**Conclusion:**

The overall observed decrease in prevalence and average duration of opioid use is encouraging. Incident prescriptions are rising in women aged 50–54 years and those with fibromyalgia while remaining steady in women aged 55–79 years. Understanding the impact of menopause and post-menopause on opioid use trends is important for effective opioid stewardship.

## How this fits in

General population studies indicate that women of menopausal and postmenopausal age are more likely to be prescribed opioids for chronic non-cancer pain (CNCP) than men or younger women, elevating their risk of opioid-derived adverse health outcomes. Research has shown that menopausal and postmenopausal women, who have much lower endogenous oestrogen levels than pre and perimenopausal women, experience linearly increasing musculoskeletal pain with age. Understanding opioid trends in these women is important, since loss of oestrogen during this phase of life increases the risk of developing adverse health conditions, such as cardiovascular disease, conditions for which long-term opioid use also increases risk. This study provides new insight into the prescription rates of opioids for CNCP in women of menopausal and postmenopausal age who have been diagnosed with a musculoskeletal condition in the UK, the understanding of which is important both for informing ongoing UK opioid stewardship efforts and future research on the safety of opioid prescribing in this population.

## Introduction

Women who have undergone menopause^1^ are, owing to diminished oestrogen levels, prone to developing severe musculoskeletal pain, often associated with chronic musculoskeletal conditions such as osteoarthritis (OA) and osteoporosis (OP).^2–5^ Research has shown that these women experience increasing pain severity with age^3^ and are consistently among the highest users of opioids for CNCP,^6–8^ putting them at greater risk of opioid misuse, opioid-related disability and mortality, and adverse health outcomes such as gastrointestinal disorders and cardiovascular diseases.^9^

Guidance from the National Institute for Health and Care Excellence (NICE) recommends against opioid use for management of primary CNCP and rheumatoid arthritis, and discourages long-term use in OA,^10–12^ given the potential for dependency, addiction, adverse health outcomes, and waning effectiveness over time.^13–15^ Even so, opioids continue to be widely prescribed for CNCP in the UK, with prescribing responsibility often falling to primary care healthcare professionls (HCPs), as is the case with other long-term conditions and menopausal symptoms in general.^6,16–19^ Recent studies have described a need for improvement in medical care for menopausal women,^19,20^ and recordings of menopause in patient health records and attribution of symptoms, such as pain, to menopause is limited.^7,21^ Recent survey data describe how many primary care HCPs find the pressure to prescribe opioids difficult to counter when faced with a patient in severe pain, even though they understand the need to limit opioid use.^22^ Thus, many continue to prescribe opioids for CNCP despite ongoing efforts by the UK Medicines and Healthcare products Regulatory Authority to curtail their usage.^23–26^

To date, no study has focused explicitly on opioid prescribing for CNCP in women of menopausal and postmenopausal age. This study aims to examine opioid prescribing trends and duration of use in these women experiencing CNCP associated with commonly diagnosed musculoskeletal conditions, using UK electronic primary healthcare data.

## Method

### Study design and study population

This drug utilisation study was conducted using the IQVIA Medical Research Data (IMRD) UK, which incorporated data supplied by The Health Improvement Network, a proprietary database of Cegedim SA. This database contains pseudonymised primary care data from >18 million patients cumulatively throughout the UK from 1994–2022, representing around 6% of the active population.

The study population comprised all women within the IMRD UK database aged 50–79 years diagnosed with CNCP during the study period of 1 January 2010–30 November 2021. CNCP was defined by formal diagnoses of specific pain-causing musculoskeletal conditions that are chronic in nature: OA, OP, rheumatoid arthritis, polymyalgia rheumatica, and fibromyalgia. The age range chosen corresponds to the range used to define menopausal and postmenopausal women in the seminal Women’s Health Initiative study, which set age limits to exclude perimenopausal women and women with pain from other conditions of advancing age.^27^ Palliative care patients, defined by palliative care or admission to hospice recordings, receiving opioids for end-of-life pain management were excluded from the study. Read codes were utilised to code for chronic non-cancer pain and palliative care (see Supplementary Tables S1 and S2).

Women entered the cohort on the date of first musculoskeletal diagnosis after their 50th birthday between 2010 and 2021. Women receiving an opioid prescription within 6 months before entry into the study were excluded. Women were censored on the earliest of: 80th birthday, transfer out of the practice, death, or the last primary care practice collection date. To calculate incident rates, women had to be registered at a general practice for at least 6 months before the study entry date to be eligible for inclusion.

### Exposure

Opioid medications included were buprenorphine, tramadol, morphine, oxycodone, codeine, co-codamol, co-dydramol, dihydrocodeine, and fentanyl.^16,28^ Oral, topical, and transdermal-formulated opioids were included.^29^ Drug code lists for opioids were generated by screening the *British National Formulary* drug dictionary legacy chapter and the Multilex drug codes (040702)^30,31^ (see Supplementary Table S3).

### Statistical analysis

Descriptive statistics were used to describe characteristics, comorbidities, and common co-medications in women using opioids with musculoskeletal conditions.^3,14^

The number of menopausal and postmenopausal women receiving their first opioid prescription (incident rate of prescribing) was calculated annually using person–years at risk (PYAR) as the denominator. A first prescription defines the initial opioid prescription after musculoskeletal diagnosis for the study participant. PYAR was calculated by summing the total amount of time each woman in the cohort was at risk of receiving their first opioid prescription while accounting for deaths, last practice collection date, those who transferred out of the practice, and a woman’s 80th birthday.^32^ Annual incident prescribing rates were reported per 1000 PYAR with 95% confidence intervals (CIs), estimated using Poisson distribution. Annual incident prescribing rates were stratified by 5-year age bands, pain indication, and Townsend score, which is a measure of socioeconomic deprivation.^33^ The prescribing prevalence for opioids was calculated using mid-year population estimates as the denominator. The mid-year population was estimated by calculating the number of menopausal and postmenopausal women each year on 1 July in the database. The annual prevalence was expressed per 100 women and stratified by opioid type in a post-hoc analysis. Relative changes in the yearly incident and prevalence rates were expressed as percentages, and the average annual percentage change in opioid prescribing throughout the study period was assessed using linear regression.^34^

Discontinuation rates were calculated using Kaplan–Meier curves. Prescriptions refilled within 6 months were used to define women having multiple opioid treatment episodes. Rates were further stratified by specific calendar year intervals:<2012, 2012–2017, and >2017. Intervals were chosen based on changes in prevalence data. All analyses were conducted using Stata (version 17).

## Results

From 2010–2021, 340 085 menopausal and postmenopausal women aged 50–79 years with CNCP contributed person–years to this study. These women received 11 309 603 opioid prescriptions in total. The mean age of musculoskeletal condition diagnosis in these women was 67.7 years, with a standard deviation (SD) of 7.9 years. Osteoarthritis was the most common diagnosis among the cohort, accounting for 48.9% of diagnoses, compared to other musculoskeletal conditions analysed. The proportion of women receiving opioids increased with age, increasing from 7.1% in women aged 50-54 to 24.7% in those aged 75-79 years. Table 1 provides the characteristics of the study population.

**Table 1.** Study population characteristics

Characteristic	Opioid users
**Total,** ***n***	340 085
**Mean age,^a^ years (SD)**	67.7 (7.9)
**Age group, years, *n* (%)**	
50–54	24 257 (7.1)
55–59	39 582 (11.6)
60–64	52 138 (15.3)
65–69	64 801 (19.1)
70–74	75 446 (22.2)
75–79	83 861 (24.7)
**Pain indications, *n* (%**)	
Rheumatoid arthritis	34 067 (10.0)
Osteoarthritis	166 170 (48.9)
Osteoporosis	94 121 (27.7)
Polymyalgia rheumatica	18 359 (5.4)
Fibromyalgia	27 368 (8.0)
**Townsend score,^b^ *n* (%**)	
1	71 652 (21.1)
2	67 156 (19.7)
3	62 507 (18.4)
4	54 506 (16.0)
5	38 254 (11.2)
’Missing’	46 010 (13.5)
**Number of opioid prescriptions, *n* (%**)	
1	189 997 (55.9)
2–5	127 674 (37.5)
6–10	15 263 (4.5)
11–25	5682 (1.7)
26–50	1020 (0.3)
>50	449 (0.1)
**Concomitant medications, *n* (%)^c^**	
Hormone replacement therapy	8731 (2.6)
Non-steroidal anti-inflammatory drugs	54 925 (16.2)
Cyclooxygenase-2 inhibitors	5165 (1.5)
**Comorbidities, *n* (%**)	
Depression	79 378 (23.3)
Obesity	43 867 (12.9)
Diabetes	44 431 (13.1)
Stroke	21 605 (6.4)
Myocardial infarction	7274 (2.1)
Venous thromboembolism	30 178 (8.9)
Vasomotor symptoms	37 256 (11.0)

^a^Age at first musculoskeletal diagnosis. ^b^Socioeconomic deprivation score: 1 = least deprived, 5 = most deprived. ^c^No users of combination pregabalin and gabapentin were identified. SD = standard deviation.

### Prescribing incidence of opioid medications

**Figure 1.**
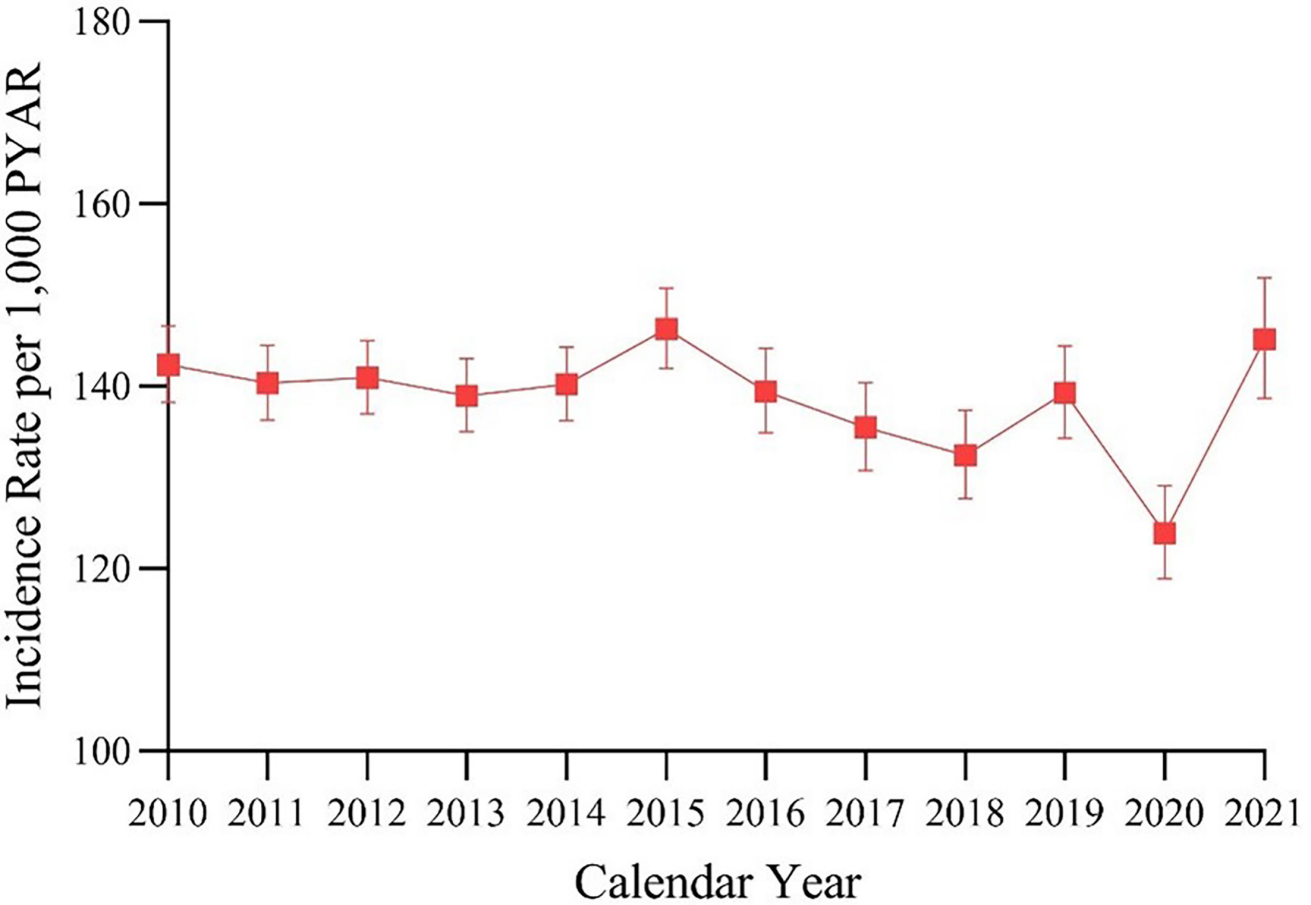
Annual crude incidence rate of opioid prescribing in the menopausal and postmenopausal population with chronic non-cancer pain from 2010–2021. PYAR = person–years at risk.

From 2010–2021, new opioid prescription rates in the study population remained relatively constant with the exception of 2020 (Figure 1). The annual incidence rate (IR) of new opioid prescriptions changed from 142.4 (95% CI = 138.2 to 146.7) per 1000 PYAR in 2010 to 139.3 (95% CI = 134.3 to 144.4) per 1000 PYAR in 2019. The IR decreased to 123.9 (95% CI = 118.9 to 129.1) per 1000 PYAR in 2020 and then increased to 145.2 (95% CI = 138.7 to 151.9) per 1000 PYAR in 2021. The temporary dip in 2020 could be owing to prescribing difficulty during COVID-19 lockdowns.^7^ Using linear regression, the average annual percentage change in opioid incidence was -0.5% (95% CI = -1.3 to 0.3).

**Figure 2.**
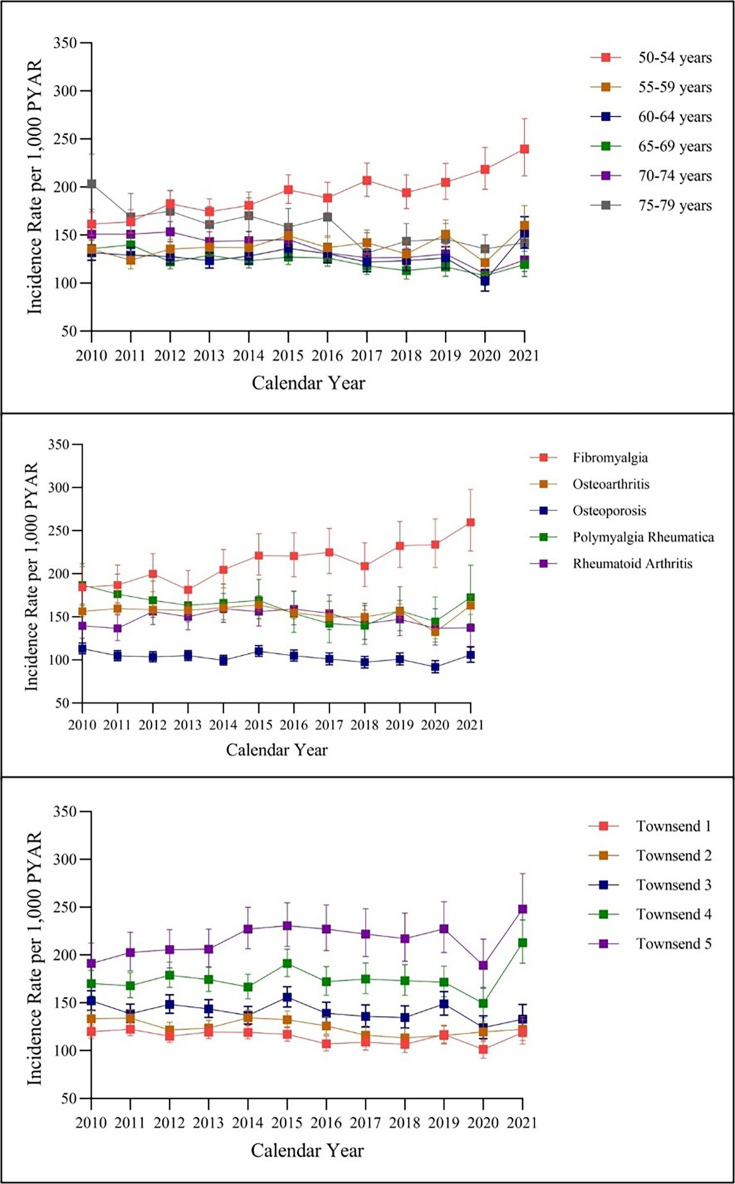
Annual incidence rate of opioid prescriptions stratified by age group, pain condition, and Townsend score in the menopausal and postmenopausal population with chronic non-cancer pain from 2010–2021. PYAR = person–years at risk.

Stratification by age showed that annual opioid prescription IRs were highest in women aged 50–54 years from 2010 to 2021: from 161.4 (95% CI = 149.7 to 174.0) per 1000 PYAR in 2010 to 239.6 (95% CI = 211.7 to 271.2) per 1000 PYAR in 2021. The average annual percentage change for the age group 50–54 years, 3.1% (95% CI = 2.3 to 3.9), showed an overall increase in new opioid prescriptions from 2010–2021 (Figure 2).

Results stratified by pain indication showed women with fibromyalgia received the highest number of new opioid prescriptions. New opioid prescriptions for fibromyalgia steadily increased from 2010 (184.4 [95% CI = 163.3 to 208.3] per 1000 PYAR) to 2021 (259.6 [95% CI = 226.4 to 297.8] per 1000 PYAR). The average annual percentage change indicated an increased rate of new opioid prescriptions, 2.8% (95% CI = 1.8 to 3.7), for fibromyalgia pain during the study period. Menopausal and postmenopausal women with OP consistently received the fewest new opioid prescriptions from 2010–2021 (annual percentage change -0.9% [95% CI = -1.7 to 0.01]) (Figure 2).

Results stratified by Townsend score showed that women from the most socioeconomic deprived areas (Townsend score 5) consistently had the highest incident rates of opioid prescriptions from 2010–2021, with a peak of 248.2 (95% CI = 216.0 to 285.2) per 1000 PYAR in 2021. Women from the least deprived areas (Townsend score 1) had the lowest annual IRs. These results were consistent across all age ranges studied. The average annual percentage change in opioid prescribing rates remained constant across all Townsend groups (Figure 2).

### Prescribing prevalence of opioids

**Figure 3.**
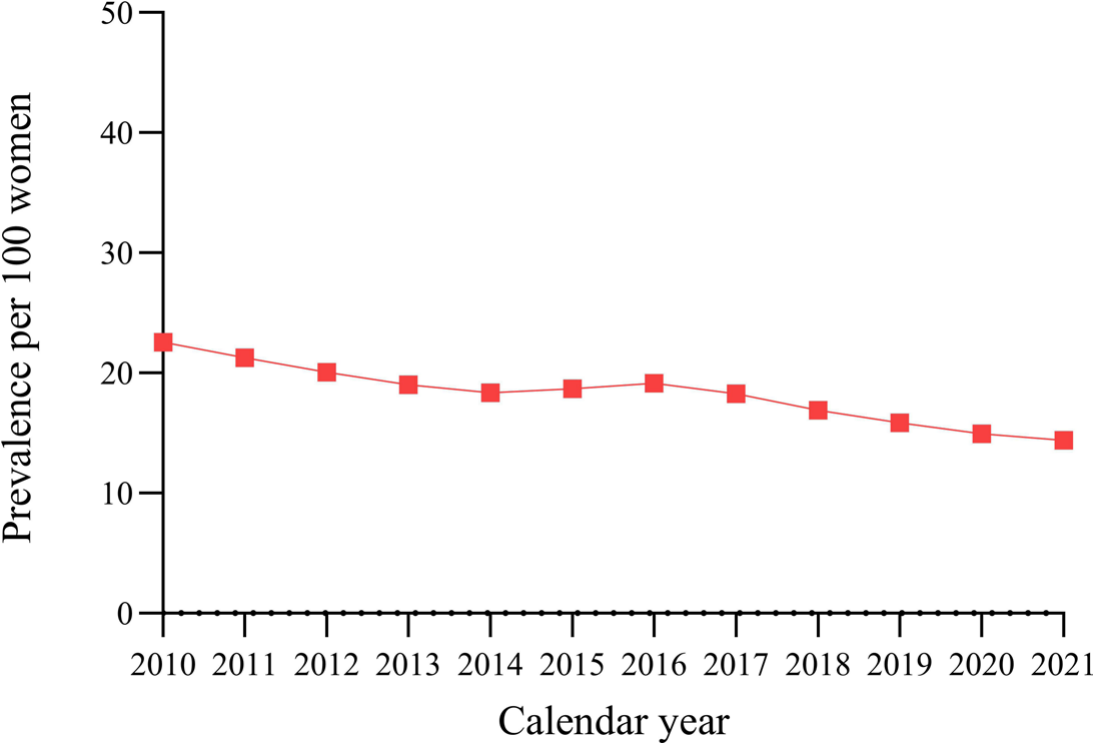
Annual opioid prescribing prevalence in the menopausal and postmenopausal population with chronic non-cancer pain from 2010–2021

The annual prescribing prevalence of opioids decreased during the study period, from 23% (2010) to 14% (2021), with an average annual percentage change of -3.9% (95% CI = -4.6 to -3.2) (Figure 3). Co-codamol and tramadol had the highest prescribing prevalence during the study period (see Supplementary Figure S1).

### Discontinuation rates of opioid prescriptions

**Figure 4.**
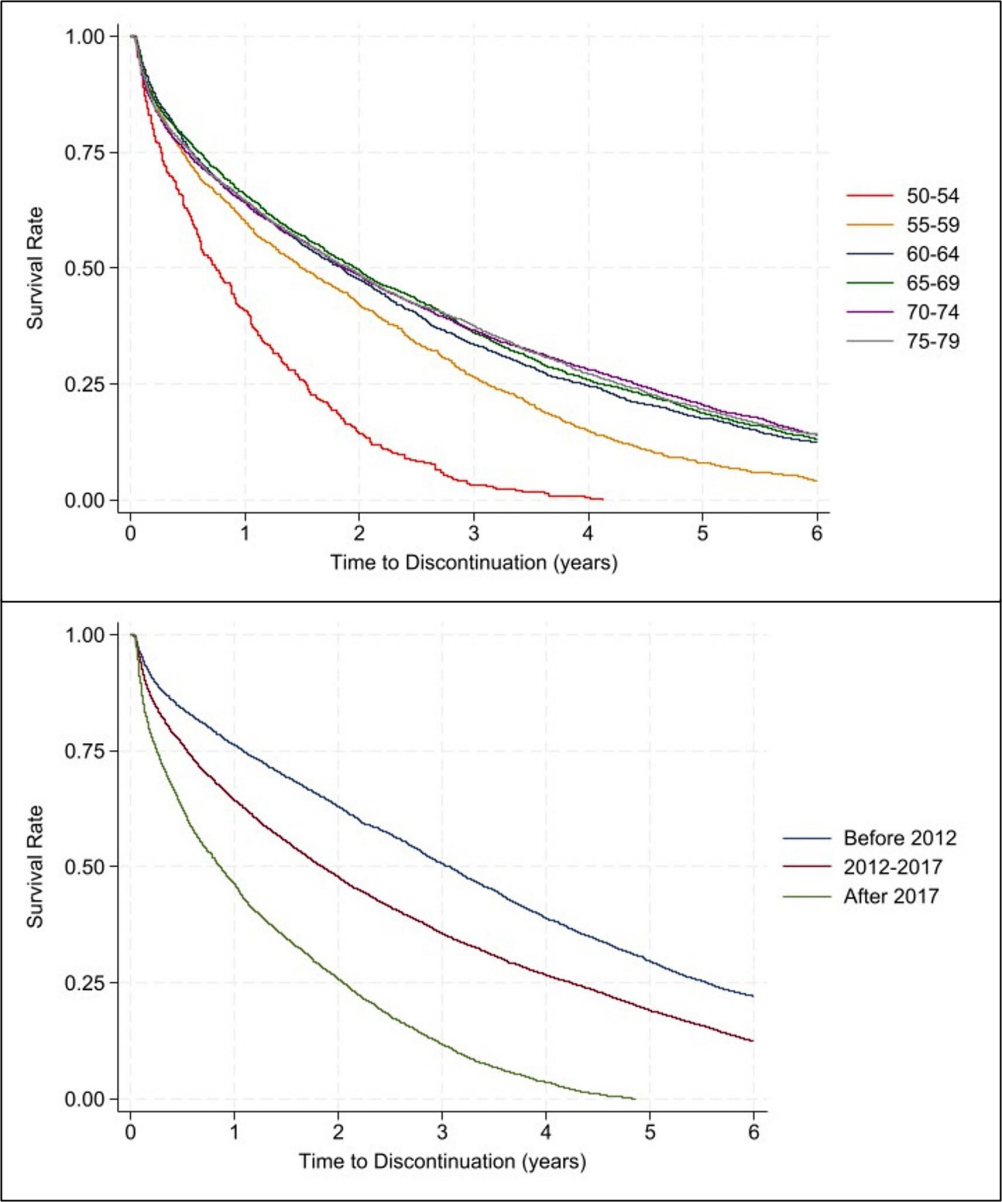
Discontinuation rates of opioid prescriptions stratified by age group and calendar year in the menopausal and postmenopausal population with chronic non-cancer pain from 2010–2021. agegrp = age group.

The average duration of opioid use steadily declined over the study period (2010-2021), decreasing from 3 years in 2010 to 1 year after 2017 (Figure 4). Women discontinued opioids more quickly post-2017, with 50% being prescribed opioids for less than 12 months (1 year).

Discontinuation rates were similar (~2 years) across pain indications and Townsend scores from 2010–2021 (see Supplementary Figures S2 and S3). Women aged 50–54 years (and aged 45–49 years; see Supplementary Figure S4) were prescribed opioids for a shorter duration (<1 year) compared with older age groups (~2 years) (Figure 4).

## Discussion

### Summary

New opioid prescription rates for menopausal and postmenopausal women aged 50–79 years with musculoskeletal-derived CNCP remained relatively steady between 2010 and 2021, with the exception of 2020; however, overall annual opioid prescribing prevalence in this population decreased from 23% (2010) to 14% (2021). Our discontinuation analysis showed a steadily decreasing average duration of opioid use with time. Larger numbers of women discontinued opioids within ~1 year after 2017, compared with ~2 years from 2012–2017 and ~3 years before 2012. Overall, use of opioids increased with age, as expected. Women with OA, a condition for which guidelines include limited opioid use,^2^ represented the greatest proportion of opioid users (Table 1).

Women aged 50–54 years discontinued opioids most quickly (<1 year) but also had the highest incident prescribing rates and greatest annual increases in new prescriptions; a sensitivity analysis in women aged 45–49 years showed that women in this age group discontinued even faster (see Supplementary Figure S4). Incident prescribing rates for older women (55–79 years) did not rise but remained steady over time and their duration of opioid use was longer, remained steady (~2 years), and did not decline as it did in younger women.

Women diagnosed with fibromyalgia had increasingly higher numbers of new opioid prescriptions from 2010–2021 compared with other conditions studied. In general, IRs of opioid prescribing increased with increasing Townsend score.

### Strengths and limitations

A strength of our study is the utilisation of electronic healthcare data that compiles general practice data from across the UK. This allowed for the creation of a UK-wide representative sample of menopausal and postmenopausal women using opioids with CNCP. However, IMRD UK only records prescription data and lacks information on prescription redemption or patient intake status. Additionally, secondary care prescribing is not captured by the IMRD UK data, potentially leading to an underestimation of opioid prescribing.

Our reliance solely on age-based criteria to define a menopausal and postmenopausal cohort might exclude some women who attain natural menopause at younger ages; however, limiting the lower age range to 50 years rather than using the lower limit of the World Health Organization (WHO) average menopausal age range^1^ enables exclusion of more women who might be perimenopausal rather than menopausal and postmenopausal in our analyses. This is important since oestrogen levels, which impact pain sensitivity and intensity,^35^ greatly differ in these women.^36–38^ Finally, our study aggregated opioids for trend analyses and did not focus on trends based on potency or doses of prescribed opioids; this would be useful in future studies to further understand risks within the population studied.

CNCP from diagnosed musculoskeletal conditions was considered in this study. Excluding pain resulting from non-specific diagnoses such as low back pain, which can be neuropathic in nature, may have resulted in an underestimation of opioid prescribing for musculoskeletal-derived CNCP.

### Comparison with existing literature

To the best of our knowledge, this is the first UK-based opioid trend study conducted in menopausal and postmenopausal-aged women diagnosed with musculoskeletal-derived CNCP. A recent study found that in patients prescribed opioids for rheumatic and musculoskeletal diseases, the majority being women aged ≥45 years, prevalent opioid user trends decreased post-2017 while new user trends remained relatively stable, consistent with our study findings.^7^ Within general population studies, women aged ≥45 years have consistently been the largest opioid user group for CNCP.^6–8,39^ Women in all age groups studied used opioids for prolonged durations, with duration of use increasing with age. This finding is consistent with prior UK primary care patient data analyses, which showed women aged ≥50 years not only used opioids longer-term than younger women or men but also increased their duration of use significantly when aged >65 years.^40^

Across all age ranges studied, menopausal and postmenopausal women with greater relative socioeconomic deprivation have a greater likelihood of being prescribed opioids for CNCP. This finding is consistent with prior research showing more frequent opioid prescribing within UK primary care practices located in regions of the UK with high socioeconomic deprivation.^41^

### Implications for research and practice

Our study shows new opioid prescription rates are rising in women aged 50–54 years while remaining steady in women aged ≥55 years; these younger women also discontinue opioid use faster than older women. Given the age-based criteria used to define our study cohort, it may be that a considerable proportion of women aged 50–54 years are perimenopausal rather than menopausal and postmenopausal. A sensitivity analysis extending the lower age to 45 years showed similar trends, supporting this assumption (see Supplementary Figures S5–S7). The widely fluctuating oestrogen levels observed in perimenopausal women particularly predispose them to development of musculoskeletal pain, which can be sudden and intense in nature, albeit often transient.^3,37,38^ The incidence and discontinuation trends observed in women aged 50–54 years, and 45–49 years, compared with women aged ≥55 years are unsurprising, given menopausal and postmenopausal women are known to have steady, low oestrogen levels,^36^ which underpin age-related pain increases; these steady, age-related pain increases result in the observed steady rates of prescribing and longer duration of overall use compared with younger women, consistent with prior general population studies in patients aged ≥65 years.^10,12,42^

Given the trends observed for women aged<55 years in our study, consideration should be given to the possibility that their musculoskeletal pain is owing to the natural musculoskeletal syndrome of menopause^21^ rather than specifically diagnosed musculoskeletal conditions. The observed increasing incident rate of opioid prescribing for fibromyalgia supports this possibility, since this syndrome has similar presentation to the climacteric.^43^ Greater support for primary healthcare practitioners in diagnosing and treating symptoms attributable to menopause could help lessen opioid use.^19,20^ At a minimum, we believe that recording of menopausal status in medical records should become a standard to assist future research in this population.

Our results show that women with greater socioeconomic deprivation are more likely to be prescribed opioids, consistent with prior research.^16,44^ These patients may be less able to seek alternative treatments owing to work obligations, higher costs of alternative treatments, or insufficient available healthcare resources.^22,29,45^ Better overall healthcare options and increased medical resources, including for women’s health concerns, are particularly needed in socioeconomically deprived areas if UK opioid stewardship efforts are to succeed.

Finally, to fully realise the desired outcomes of ongoing efforts to curtail opioid use, there needs to be greater focus on effective pain management in menopausal and postmenopausal women generally. This population is continually among the highest users of opioids,^6–8^ often at high doses,^9^ yet little research exists to fully understand the long-term health risks this poses for these women, especially considering their increased likelihood of polypharmacy, comorbidities, frailty, and risks from alternative pain medications, such as non-steroidal anti-inflammatory drugs, as they age. More research on the risks of long-term opioid prescribing is needed to assist primary care practitioners to balance the risks of opioid use with the difficulty of finding suitable and effective alternative treatments for menopausal and postmenopausal women presenting with severe pain.^40,46,47^

## Data

Study data are not available; however, IMRD UK data can be requested from IQVIA (https://www.iqvia.com).
